# Complete genomic sequence of Epstein-Barr virus in nasopharyngeal carcinoma cell line C666-1

**DOI:** 10.1186/1750-9378-8-29

**Published:** 2013-08-02

**Authors:** Ken Kai-Yuen Tso, Kevin Yuk-Lap Yip, Cathy Ka-Yan Mak, Grace Tin-Yun Chung, Sau-Dan Lee, Siu-Tim Cheung, Ka-Fai To, Kwok-Wai Lo

**Affiliations:** 1 Department of Computer Science and Engineering, The Chinese University of Hong Kong, Hong Kong SAR, China; 2 Department of Anatomical and Cellular Pathology, State Key Laboratory in Oncology in South China, Prince of Wales Hospital, The Chinese University of Hong Kong, Hong Kong SAR, China; 3 Li Ka Shing Institute of Health Science, The Chinese University of Hong Kong, Hong Kong SAR, China; 4 Department of Surgery, University of Hong Kong, Hong Kong SAR, China

**Keywords:** Epstein-Barr virus, Nasopharyngeal carcinoma, Whole-genome deep sequencing, Single-nucleotide variations, Indels, Phylogenetic analysis, BNRF

## Abstract

**Background:**

Nasopharyngeal carcinoma is a distinct type of head and neck cancer which is consistently associated with Epstein-Barr virus (EBV). The C666-1 cell line is the only in vitro native EBV-infected NPC cell model commonly used for study of the viral-host interaction. Nevertheless, the complete EBV genome sequence in this in vitro EBV-infected NPC model has not been characterized.

**Objective:**

To determine the complete EBV genome sequence in C666-1 cells.

**Methods:**

The C666-1 genome was sequenced by 100-bases pair-end massive parallel sequencing. Bioinformatics analysis was performed to extract the EBV sequences and construct an EBV consensus sequence map. PCR amplification and Sanger DNA sequencing were used for sequence validation and gap filling. A phylogenetic analysis of EBV strain in C666-1 cells and other reported EBV strains was performed.

**Results:**

A 171,317 bp complete EBV genome of C666-1 was successfully constructed (GenBank accession number: KC617875). Phylogenetic analysis of EBV genome in C666-1 revealed that the C666-1 EBV strain is closely related to the reported strains in NPC primary tumors.

**Conclusion:**

C666-1 contains a representative NPC-associated EBV genome and might serve as an important model for studying the roles or function of viral proteins in NPC tumorigenesis.

## Findings

NPC is a distinct type of head and neck cancer which is consistently associated with Epstein-Barr virus (EBV). Detection of clonal EBV genome in both precancerous lesions and invasive cancers indicates that EBV latent infection is an early event in the tumorigenesis of NPC. Since we established the EBV-positive NPC cell line C666-1 and reported it about fifteen years ago, it has been widely used for investigating host-viral interaction, elucidating the function and transcriptional regulation of EBV-encoded latent genes and miRNAs, and developing EBV targeting therapeutic strategies [1]. The origin of this cell line was from an undifferentiated NPC biopsy of a Hong Kong patient [1]. It contains normal episomal EBV genome and shows latency II EBV gene expression pattern. A number of studies demonstrated the distinct NF-κb, STAT3, AKT and NOTCH pathways in this cell line as well as the in vivo samples including EBV-positive NPC xenografts (e.g., C15, C17, xeno-2117) and primary tumors [2]. Recently, two novel EBV-encoded microRNAs, miR-BART21 and miR-BART22 have been discovered from this EBV-positive epithelial cell line [3].

**Figure 1:**
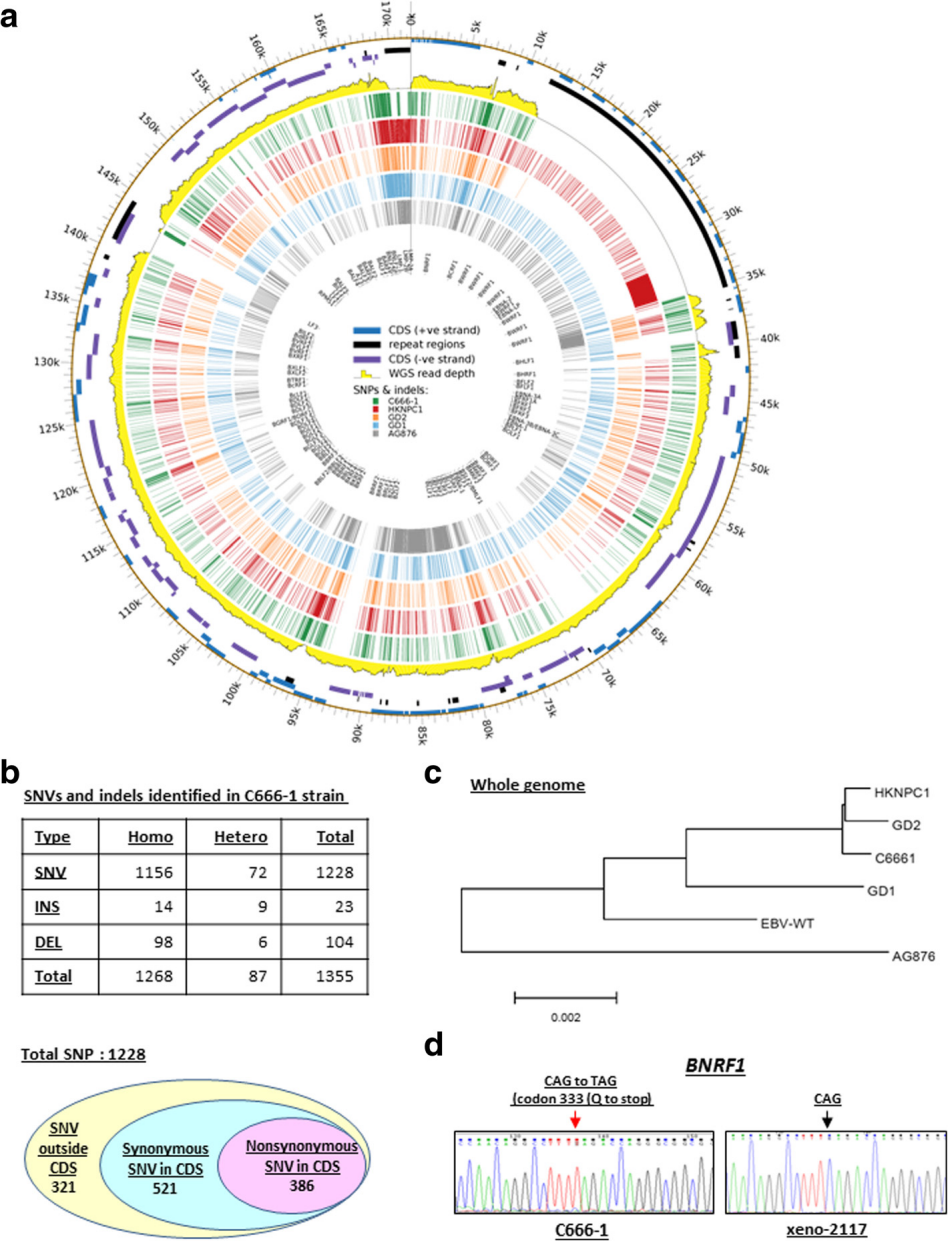
**Characterization of the EBV genome sequence derived from whole-genome deep sequencing of NPC cell line C666-1. (a)** Circos plot demonstrates the genome-wide comparison of SNVs and indels in EBV genome of C666-1 (green bars) and those of other reported strains (HKNPC1, red bars; GD2, orange bars; GD1, blue bars; AG876, grey bars). The WT-EBV genome sequence was used as reference. **(b)** Summary of SNVs and indels identified in C666-1 strain. **(c)** Phylogenetic analysis of the genome sequences in five EBV strains, C666-1, HKNPC1, GD1, GD2, AG876 and EBV-WT. **(d)** A nonsense mutation in codon 333 (Q to stop) of *BNRF1* identified in the C666-1 strain. The wild type sequence from the NPC xenograft xeno-2117 is also shown.

Despite C666-1 being the only in vitro native EBV-infected NPC model worldwide, the EBV genome in this cell line has not been fully characterized until now. To facilitate the EBV-related studies using this unique cell line, we constructed the EBV genome map through bioinformatic analysis and experimental validation of our recent whole-genome deep sequencing results (Additional file 1 Supplementary methodology). By 100-base pair-end genomic sequencing on Illumina HiSeq 2000 genome sequencer, the C666-1 genome was sequenced with average >75-fold coverage as described [4]. A total of 2,511,210,660 reads (251 Gb) were collected from the sample. By using an approach that combines the results of two alignment strategies, namely aligning the reads to both human and EBV reference genomes (EBV-WT; GeneBank accession number AJ507799) at the same time, and aligning them first to the human genome and then the remaining reads to the EBV reference genome, we extracted a total of 857,595 kb EBV sequences from the collected C666-1 data. A high coverage value of 504 folds to EBV genome was yielded. All uniquely mapped EBV sequences were assembled into a 143,734 bpconsensus sequence with a read depth of at least 10 reads. We validated the poorly aligned and questionable regions and filled up the gaps by PCR amplification and conventional Sanger DNA sequencing. The regions failed to be assembled (e.g. with highly repetitive sequences) are represented by tracts of Ns as described previously [5]. A 171,317 bp complete EBV genome of C666-1 was constructed (Figure 1a). This newly assembled C666-1 EBV sequence was submitted to GenBank with accession number KC617875. The study was approved by the University Animal Experimentation Ethics Committee (AEEC) (13-036-MIS) of the Chinese University of Hong Kong.

**Figure 2:**
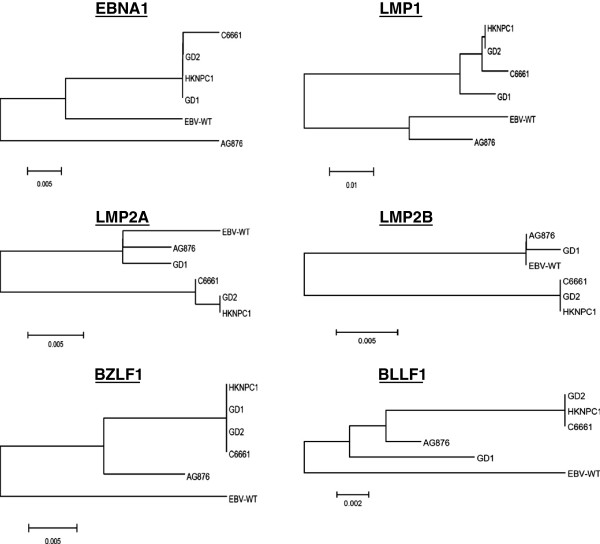
Phylogenetic analysis of EBNA1, LMP1, LMP2A, LMP2B, BZLF1 and BLLF1 protein sequences of C666-1 and other reported EBV strains (HKNPC1, GD1, GD2, AG876 and WT-EBV).

In this study, we have assembled the EBV genome in C666-1 using high-coverage genome sequencing data. Since no PCR amplification was involved, both homogenous and heterogeneous genome variations are accurately determined. Comparing with the EBV-WT reference genomic sequence (AJ507799), we have revealed a total of 1,268 homogenous and 87 heterogeneous sequence variations. These changes include 127 indels and 1,228 SNVs. Among the SNVs, 907 are located within the coding regions and 41.3% (386/907) of them are nonsynonymous (Figure 1b). The sequence variations in selected SNVs were confirmed by Sanger DNA sequencing. Phylogenetic analysis of whole EBV genomes in C666-1 and the reported strains (EBV-WT, AG876, GD1, GD2, and HKNPC1) showed that C666-1 is closely related to the GD2 and HKNPC1 strains (Figure 1c) [5,6]. It has great divergence with the AG876 and reference EBV-WT genome. Similar results were observed when we compared the protein sequences of various EBV lytic (BZLF1, BLLF1) and latent (EBNA1, LMP1, LMP2) genes (Figure 2). A number of studies have also shown that BZLF1 and LMP1 sequences of the isolates from Hong Kong NPC patients are distinct from that of the EBV-infected lymphoid cells derived in Africa or Western countries [7]–[9]. The findings imply that C666-1 might serve as an important model for studying the roles or functions of viral proteins in NPC tumorigenesis. Among the four EBV strains from South China, the isolate from NPC patient’s saliva (GD1) shows the greatest divergence with those from the tumors (C666-1, GD2, HKNPC1). This finding suggests the presence of tumor-associated EBV strain(s) in NPC patients. Nevertheless, a comprehensive sequencing of EBV isolates from saliva, peripheral blood and tumor specimens in a panel of NPC patients may prove this hypothesis. A summary of non-synonymous SNVs in the majority of EBV-encoded lytic and latent genes of C666-1 strain versus those of GD2 and HKNPC1 is shown in Additional file 2: Table S1. In the latent genes including EBNA1, EBNA3B/3C, LMP1 and LMP2B genes, high frequencies of C666-1 specific non-synonymous SNVs were observed. The prevalence and function of these SNVs in NPC need further elucidations. Previously, we have demonstrated that multiple EBV-encoded BART miRNAs (miR-BART1-5p, miR-BART16 and miR-BART17-5p) target the 3′UTR of the LMP1 gene [10]. The predicted target sequences of these 3 EBV-encoded BART miRNAs in the 3′UTR of the LMP1 gene are highly conserved in the NPC-derived EBV strains. In this study, we also found no polymorphism in the predicted target sequences of the miR-BART1-5p, 16, and 17-5p in the C666-1 EBV strain.

Apart from the missense mutations, a homogenous nonsense mutation in the lytic gene, BNRF1, which encodes an EBV major tegument protein was found. We confirmed the mutation in C666-1 by PCR amplification and Sanger Sequencing (Figure 1d). This finding indicates the deficiency of BNRF1 protein expression in this in vitro EBV-positive NPC models. Notably, it was reported that EBV with BNRF1 deletion also showed efficient lytic replication and production of mature viral particles. There are no major structural alterations in the BNRF1-deleted virus [11]. Further elucidation of the virus production and lytic cycle of this BNRF1-deficient C666-1 strain is needed. On the other hand, a recent study has reported that BNRF1 activates viral early gene BZLF1 transcription via disrupting cellular DAXX-ATRX in 293 cells. Thus, BNRF1 deficiency may help to maintain the latent EBV genome in NPC cells [12]. On the other hand, loss of BNRF1 in the C666-1 strain may impact the escape from the host immune responses in the NPC patients since BNRF1 is a defined target of the EBV-specific T-helper-cell response.

In summary, we delineated the whole EBV genome sequence in C666-1, which might serve as an important resource for NPC studies. The phylogenetic analysis indicates the C666-1 strain as a representative strain for EBV-associated NPC.

## Abbreviations

EBV: Epstein-Barr virus; NPC: Nasopharyngeal carcinoma; Indels: Insertions/deletions; SNVs: Single-nucleotide variations; CDS: Coding sequence.

## Competing interests

The authors declare that they have no competing interests.

## Authors’ contributions

KWL and KYLY designed the study; KWL, KYLY, and KFT drafted the manuscript; KKYT, KYLY and SDL participated in the bioinformatics analysis and sequence alignment; CKYM, GTYC, STC carried out the molecular genetic studies. All authors read and approved the final manuscript.

## Authors’ information

Ken Kai-Yuen Tso and Kevin Yuk-Lap Yip are co-first authors.

## Supplementary Material

Additional file 1 Supplementary methodology. Click here for file

Additional file 2: Table S1 Non-synonymous mutations and amino acid changes commonly found in NPC tumor samples (C666-1, HKNPC1 and GD2). Click here for file
