# Supplementary material for: Complete genomic sequence of Epstein-Barr virus in nasopharyngeal carcinoma cell line C666-1
Source: Infect Agent Cancer. 2013 Aug 2;8:29. doi: 10.1186/1750-9378-8-29 (PMC3734220; doi:10.1186/1750-9378-8-29)
Supplement: Additional file 1 — Supplementary methodology. [file 1750-9378-8-29-S1.docx]

**Supplementary methodology**

**Whole-genome sequencing**: The sequencing library was generated from genomic DNA extracted from C666-1 cells according to the standard protocol of Illumina Inc. for 100 -based paired-end sequencing. The samples were than sequenced using Illumina HiSeq2000 instrument as described (ref. 4).

**Determining the EBV genome**: We used an alignment-based approach to determine the sequence of the EBV genome in the C666-1 cells. We used two different strategies to perform the read alignments, and combined their results based on the confidence of their alignment quality. In the first strategy, we aligned all the reads to both the human reference genome (hg19) and the EBV-WT genome at the same time using the fast aligner BWA (PMID: 19451168, version 0.6.2-r126). In the second strategy, we first aligned all the reads to the human reference genome using BWA, and then aligned the unaligned reads only to EBV-WT using a more sensitive aligner SHRiMP2 (PMID: 21278192, version 2.2.2). The first strategy ensures that each read is aligned to the most similar region in the two genomes, while the second strategy provides a more rigorous filtering by discarding any reads with high similarity to a region in the human genome. For both strategies, we post-processed the alignment results by removing duplicated reads using SAMtools (PMID: 19505943), performing local re-alignment using GATK (PMID: 20644199) and calculating Base Alignment Quality (BAQ) scores using SAMtools. Subsequently, a list of genetic variants between the C666-1 EBV genome and EBV-WT was called by using the alignment results with SAMtools. We combined the variant lists from the two strategies by comparing their BAQ values. In general, if a genetic variant appeared confidently on one or both of the lists, defined as a variant with a BAQ score > 10 and at least 10 reads aligned to the positions, we included it in the combined list. If there was a disagreement between the two lists about a base but both were confident, we used the result from the second strategy as it is expected to cause fewer false alignments. Finally, the combined list of genetic variants was used to produce the sequence of the C666-1 EBV genome by applying the variants to EBV-WT. Any bases in the reference genome with no aligned reads, such as some repeat regions, are marked as ‘N’.

**Phylogenetic analysis**: The comparison of whole EBV genome sequence of C666-1 against HKNPC1 (GenBank accession no.: JQ009376.1), GD1 (GenBank accession no.: AY961628.3), GD2 (GenBank accession no.: HQ020558.1) , AG876 (GenBank accession no.: DQ279927.1) and WT-EBV(GenBank accession no.: NC_007605.1) was performed by first aligning the sequences using MAFFT (PMID: 23023983) with default settings, followed by the construction of a phylogenetic tree using MEGA 5 (PMID: 21546353) with the Neighbor-Joining (NJ) algorithm. The gene-based phylogenetic trees were constructed in the same way based on the protein sequences of the genes.
