# Supplementary material for: Complete genomic sequence of Epstein-Barr virus in nasopharyngeal carcinoma cell line C666-1
Source: Infect Agent Cancer. 2013 Aug 2;8:29. doi: 10.1186/1750-9378-8-29 (PMC3734220; doi:10.1186/1750-9378-8-29)
Supplement: Additional file 2: Table S1 — Non-synonymous mutations and amino acid changes commonly found in NPC tumor samples (C666-1, HKNPC1 and GD2). [file 1750-9378-8-29-S2.docx]

TableS1: Non-synonymous mutations and amino acid changes commonly found in NPC tumor samples (C666-1, HKNPC1 and GD2)

| EBV Coordinate* | Nucleotide change | Gene | Residue | C666-1 | HKNPC1 | GD2 | GD1 | B95-8 | AG876 |
| --- | --- | --- | --- | --- | --- | --- | --- | --- | --- |
| 159 | C/G | *LMP2A* | 153 | S | S | S | T | T | T |
| 207 | G/A | *LMP2A* | 169 | N | N | N | S | S | S |
| 213 | A/C | *LMP2A* | 171 | S | S | S | Y | Y | Y |
| 613 | C/G | *LMP2A* | 248 | M | M | M | I | I | I |
| 629 | G/C | *LMP2A* | 254 | L | L | L | V | V | V |
| 632 | C/G | *LMP2A* | 255 | V | V | V | L | L | L |
| 1073 | A/G | *LMP2A* | 350 | V | V | V | I | I | I |
| 1075 | C/T | *LMP2A* | 350 | V | V | V | I | I | I |
| 1134 | T/C | *LMP2A* | 370 | T | T | T | I | I | I |
| 1196 | A/C | *LMP2A* | 391 | H | H | H | N | N | N |
| 3821 | G/A | *BNRF1* | 696 | R | R | R | G | G | G |
| 5399 | G/A | *BNRF1* | 1222 | I | I | I | V | V | V |
| 36283 | T/G | *EBNA2* | 23 | R | R | R | L | L | L |
| 36668 | T/G | *EBNA2* | 151 | M | M | M | I | I | I |
| 36702 | A/G | *EBNA2* | 163 | V | V | V | R | R | R |
| 36769 | A/G | *EBNA2* | 185 | R | R | R | Q | Q | Q |
| 36799 | T/C | *EBNA2* | 195 | T | T | T | M | M | M |
| 36951 | C/A | *EBNA2* | 246 | S | S | S | R | R | R |
| 37054 | C/A | *EBNA2* | 280 | N | N | N | T | T | T |
| 42322 | G/T | *BHRF1* | 79 | L | L | L | V | V | V |
| 45489 | A/T | *BFLF1* | 250 | Q | Q | Q | Q | L | L |
| 46106 | G/C | *BFLF1* | 44 | E | E | E | E | D | D |
| 46134 | A/G | *BFLF1* | 35 | T | T | T | T | I | I |
| 46368 | C/T | *BFRF1A* | 45 | F | F | F | F | L | L |
| 46410 | C/A | *BFRF1A* | 59 | I | I | I | I | L | L |
| 47568 | T/G | *BFRF2* | 17 | G | G | G | G | C | C |
| 47620 | A/C | *BFRF2* | 34 | P | P | P | P | Q | Q |
| 47638 | G/A | *BFRF2* | 40 | E | G | G | G | E | E |
| 47905 | T/C | *BFRF2* | 129 | A | A | A | A | V | V |
| 47907 | G/T | *BFRF2* | 130 | F | F | F | F | V | V |
| 48609 | C/T | *BFRF2* | 364 | W | W | W | W | R | R |
| 48634 | A/T | *BFRF2* | 372 | L | L | L | H | H | H |
| 48904 | G/A | *BFRF2* | 462 | Q | Q | Q | Q | R | R |
| 50134 | G/C | *BPLF1* | 3036 | E | E | E | Q | Q | Q |
| 50241 | C/T | *BPLF1* | 3000 | Q | Q | Q | Q | R | R |
| 50557 | G/T | *BPLF1* | 2895 | S | S | S | S | R | R |
| 50558 | C/T | *BPLF1* | 2895 | S | S | S | S | R | R |
| 50635 | A/G | *BPLF1* | 2869 | L | L | L | L | F | F |
| 50947 | G/A | *BPLF1* | 2765 | S | S | S | P | P | P |
| 51152 | G/C | *BPLF1* | 2696 | R | S | S | R | R | R |
| 51664 | C/T | *BPLF1* | 2526 | N | N | N | D | D | D |
| 52504 | T/C | *BPLF1* | 2246 | D | D | D | D | N | N |
| 52709 | C/T | *BPLF1* | 2177 | I | I | I | I | M | M |
| 53605 | C/A | *BPLF1* | 1879 | F | F | F | V | V | V |
| 54705 | C/T | *BPLF1* | 1512 | Q | Q | Q | R | R | R |
| 57280 | A/C | *BPLF1* | 654 | A | A | A | S | S | S |
| 57296 | A/T | *BPLF1* | 848 | E | E | E | D | D | D |
| 57412 | G/T | *BPLF1* | 610 | I | I | I | L | L | L |
| 59206 | T/G | *BPLF1* | 12 | P | P | P | P | T | T |
| 59966 | T/G | *BOLF1* | 996 | R | R | R | S | S | S |
| 60028 | G/A | *BOLF1* | 975 | L | L | L | P | P | P |
| 60454 | G/T | *BOLF1* | 833 | Y | Y | Y | S | S | S |
| 60527 | T/C | *BOLF1* | 809 | G | G | G | R | R | R |
| 70501 | C/G | *BSLF2/BMLF1* | 465 | A | A | A | A | G | G |
| 71677 | C/T | *BSLF2/BMLF1* | 73 | Q | Q | Q | Q | R | R |
| 71766 | C/G | *BSLF2/BMLF1* | 43 | D | D | D | E | E | E |
| 73861 | C/T | *BSLF1* | 245 | T | T | T | T | A | A |
| 74364 | T/C | *BSLF1* | 77 | R | R | R | R | H | H |
| 74587 | C/T | *BSLF1* | 3 | T | T | T | T | A | A |
| 77452 | C/T | *BLLF1* | 805 | D | D | D | G | G | G |
| 77896 | A/C | *BLLF1* | 657 | R | R | R | I | I | I |
| 77920 | G/T | *BLLF1* | 649 | D | D | D | A | A | A |
| 79265 | C/G | *BLLF1* | 201 | Q | Q | Q | Q | E | E |
| 80841 | G/A | *EBNA3A* | 267 | I | I | I | V | V | V |
| 80920 | G/A | *EBNA3A* | 293 | N | N | N | S | S | S |
| 81111 | A/G | *EBNA3A* | 357 | A | A | A | T | T | T |
| 81417 | C/A | *EBNA3A* | 459 | T | T | T | P | P | P |
| 81723 | A/T | *EBNA3A* | 561 | F | F | F | I | I | I |
| 83171 | G/A | *EBNA3B* | 36 | E | E | E | G | G | G |
| 84414 | G/T | *EBNA3B* | 424 | N | N | N | K | K | K |
| 84884 | C/A | *EBNA3B* | 581 | N | N | N | T | T | T |
| 85225 | C/A | *EBNA3B* | 695 | K | K | K | Q | Q | Q |
| 85229 | C/T | *EBNA3B* | 696 | I | I | I | T | T | T |
| 85586 | A/G | *EBNA3B* | 815 | R | R | R | R | Q | Q |
| 85802 | C/G | *EBNA3B* | 887 | G | G | G | A | A | A |
| 85841 | A/G | *EBNA3B* | 900 | R | R | R | Q | Q | Q |
| 86795 | A/C | *EBNA3C* | 213 | H | H | H | Q | Q | Q |
| 87164 | A/C | *EBNA3C* | 336 | D | D | D | E | E | E |
| 87198 | A/C | *EBNA3C* | 348 | L | L | L | I | I | I |
| 88122 | C/G | *EBNA3C* | 656 | G | G | G | G | R | R |
| 88465 | A/G | *EBNA3C* | 769 | R | R | R | Q | Q | Q |
| 88501 | A/C | *EBNA3C* | 782 | P | P | P | H | H | H |
| 90455 | C/T | *BZLF1* | 138 | E | E | E | E | G | G |
| 90478 | A/C | *BZLF1* | 130 | L | L | L | L | F | F |
| 90554 | T/A | *BZLF1* | 105 | L | L | L | L | Q | Q |
| 91431 | G/T | *BRLF1* | 489 | K | K | K | K | Q | R |
| 94794 | G/A | *BRRF2* | 261 | N | N | N | N | D | D |
| 94957 | T/G | *BRRF2* | 315 | G | G | G | V | V | V |
| 95133 | A/G | *BRRF2* | 374 | A | A | A | A | T | T |
| 95141 | A/C | *BRRF2* | 377 | R | R | R | R | G | G |
| 95142 | G/C | *BRRF2* | 377 | R | R | R | R | G | G |
| 95167 | T/C | *BRRF2* | 385 | T | T | T | T | M | M |
| 95187 | A/C | *BRRF2* | 392 | Q | Q | Q | Q | K | K |
| 95197 | A/G | *BRRF2* | 395 | G | G | G | G | E | E |
| 95265 | T/C | *BRRF2* | 418 | R | R | R | C | C | C |
| 95292 | G/T | *BRRF2* | 427 | S | S | S | A | A | A |
| 95719 | A/T | *EBNA1* | 20 | S | S | S | S | T | T |
| 96751 | G/A | *EBNA1* | 363 | R | R | R | R | G | G |
| 96914 | A/T | *EBNA1* | 418 | L | L | L | L | H | H |
| 96976 | G/A | *EBNA1* | 439 | T | T | T | T | A | A |
| 97243 | A/G | *EBNA1* | 528 | V | V | V | V | I | I |
| 97258 | C/A | *EBNA1* | 533 | I | I | I | I | L | L |
| 97973 | G/A | *BKRF2* | 102 | S | S | S | S | G | G |
| 98006 | A/T | *BKRF2* | 113 | S | S | S | S | T | T |
| 99330 | A/C | *BKRF4* | 162 | P | P | P | P | Q | Q |
| 99351 | G/T | *BKRF4* | 169 | V | V | V | V | G | G |
| 99356 | C/A | *BKRF4* | 171 | N | N | N | N | H | H |
| 102600 | T/G | *BBRF1* | 229 | G | G | G | C | C | C |
| 103825 | A/G | *BBRF2* | 56 | V | V | V | V | I | I |
| 103885 | G/A | *BBRF2* | 76 | I | I | I | I | V | V |
| 104550 | A/C | *BBLF2/BBLF3* | 691 | M | M | M | I | I | I |
| 104555 | T/C | *BBLF2/BBLF3* | 690 | A | A | A | T | T | T |
| 104980 | C/G | *BBLF2/BBLF3* | 548 | A | A | A | G | G | G |
| 105265 | G/T | *BBLF2/BBLF3* | 496 | T | T | T | P | P | P |
| 106273 | T/C | *BBLF2/BBLF3* | 160 | V | V | V | V | M | M |
| 106293 | A/C | *BBLF2/BBLF3* | 153 | G | G | G | V | V | V |
| 112981 | A/T | *BGRF1/BDRF1* | 111 | L | L | L | Q | Q | Q |
| 115296 | A/G | *BGLF1* | 264 | P | P | P | P | L | L |
| 115411 | A/G | *BGLF1* | 226 | P | P | P | P | S | S |
| 118154 | C/T | *BDLF3* | 209 | T | T | T | T | A | A |
| 118373 | T/C | *BDLF3* | 136 | A | A | A | A | T | T |
| 118433 | C/A | *BDLF3* | 116 | S | S | S | A | A | A |
| 118562 | T/G | *BDLF3* | 73 | L | L | L | L | I | I |
| 125272 | C/T | *BcRF1* | 33 | A | A | A | T | T | T |
| 126191 | G/A | *BcRF1* | 339 | R | R | R | G | G | G |
| 127409 | T/C | *BcRF1* | 745 | R | R | R | C | C | C |
| 127992 | G/A | *BTRF1* | 193 | T | T | T | A | A | A |
| 128269 | C/A | *BTRF1* | 285 | Q | Q | Q | P | P | P |
| 134372 | A/G | *BVRF1* | 416 | V | V | V | M | M | M |
| 134730 | T/C | *BVRF1* | 535 | T | T | T | M | M | M |
| 136646 | G/T | *BVRF2* | 337 | S | S | S | S | A | A |
| 137316 | A/C | *BVRF2* | 559 | P | P | P | H | H | H |
| 155609 | C/T | *BALF5* | 227 | Q | Q | Q | R | R | R |
| 156637 | G/A | *BALF4* | 743 | V | V | V | A | A | A |
| 157568 | C/T | *BALF4* | 433 | N | N | N | D | D | D |
| 158143 | T/G | *BALF4* | 241 | T | T | T | K | K | K |
| 158481 | G/C | *BALF4* | 128 | E | E | E | D | D | D |
| 161036 | T/C | *BALF2* | 1093 | G | G | G | G | S | S |
| 162476 | T/C | *BALF2* | 613 | V | V | V | I | I | I |
| 163364 | C/T | *BALF2* | 317 | M | M | M | V | V | V |
| 167859 | C/T | *LMP1* | 335 | D | D | D | G | G | G |
| 168229 | C/T | *LMP1* | 212 | S | S | S | S | G | G |
| 168288 | C/G | *LMP1* | 192 | T | T | T | T | S | S |
| 168297 | T/G | *LMP1* | 189 | P | P | P | P | Q | Q |
| 168331 | G/T | *LMP1* | 178 | M | M | M | M | L | L |
| 168414 | T/G | *LMP1* | 150 | A | A | A | A | D | D |
| 168433 | A/T | *LMP1* | 144 | I | I | I | I | F | F |
| 168767 | A/C | *LMP1* | 84 | G | G | G | G | C | C |
| 168772 | G/C | *LMP1* | 82 | G | G | G | G | A | A |
| 168881 | C/T | *LMP1* | 46 | N | N | N | N | D | D |
| 168944 | G/T | *LMP1* | 25 | I | I | I | I | L | L |
| 168967 | C/A | *LMP1* | 17 | L | L | L | L | R | R |
| 168979 | C/G | *LMP1* | 13 | P | P | P | P | R | R |
| 169009 | T/C | *LMP1* | 3 | R | R | R | R | H | H |

*Coordinates of NC007605
